# Oxidative stress parameters in patients with ascending aortic dilatation

**DOI:** 10.3906/sag-1909-183

**Published:** 2020-08-26

**Authors:** Mehmet ERDOĞAN, Melike POLAT, Muhammet Cihat ÇELİK, Selçuk ÖZTÜRK, Serdal BAŞTUĞ, Yunus Emre ÖZBEBEK, Salim NEŞELİOĞLU, Murat AKÇAY

**Affiliations:** 1 Department of Cardiology, Ministry of Health Ankara City Hospital, Ankara Turkey; 2 Department of Cardiology, Faculty of Medicine, Yıldırım Beyazıt University, Ankara Turkey; 3 Department of Cardiology, Faculty of Medicine, University of Health Sciences, Ankara Training and Research Hospital, Ankara Turkey; 4 Department of Biochemistry, Faculty of Medicine, Yıldırım Beyazıt University, Ankara Turkey

**Keywords:** Ascending aortic dilatation, oxidative stress, thiols, ferroxidase, ischemia modified albumin

## Abstract

**Background/aim:**

This study aimed to determine plasma thiol, disulphide, and serum ischemia-modified albumin (IMA) levels and ferroxidase activity in patients with ascending aorta dilatation (AAD) in comparison to those without AAD and to evaluate the predictive value of these oxidative stress parameters for AAD.

**Materials and methods:**

This study was designed as a cross-sectional study of 184 patients who applied to our cardiology clinic. Our study population consisted of patients with AAD (n = 85) and without AAD (n = 99). A spectrophotometric method was used to determine plasma thiol, disulphide, and serum IMA levels and ferroxidase activity.

**Results:**

The native thiol and the total thiol levels were significantly higher in the control group than the AAD group (P < 0.001), whereas the disulphide and IMA levels and the ferroxidase activity were similar between the groups. The native thiol and the total thiol levels were inversely and significantly correlated with ascending aortic diameter (r = –0.38, P < 0.001; r = –0.39, P < 0.001; respectively). The left ventricle mass and the total thiol levels were independent predictors of ascending aortic diameter (β= 0.223, P = 0.02; β= –0.340, P < 0.001; respectively).

**Conclusion:**

Among oxidative stress parameters including thiols, disulphide, IMA, and ferroxidase activity, only the lower total thiol levels appear to confer a high risk for AAD development. Along with the proven diagnostic imaging methods, thiol levels may be helpful to diagnose and stratify patients with AAD.

## 1. Introduction

Ascending aortic dilatation (AAD) is a common and incidental condition, which can be fatal if unnoticed at an early stage. The enlargement of the ascending aorta is defined as aneurysm if it is over 1.5 times the normal diameter. As it is a clinically silent disease, it is difficult to assess the true prevalence and incidence of aortic aneurysms. Aortic aneurysms are associated with a rupture risk of more than 5% per year resulting in death [1]. Several mechanisms have been shown to be linked with the development of AAD such as hemodynamic force, destructive remodeling of the extracellular matrix, familial predisposition, transmural inflammation of the aorta, and oxidative stress [2–5]. Particularly, the imbalance between matrix metalloproteinases (MMPs) and their inhibitors plays an important role in the degradation of the structure of aortic wall and the development of aortic aneurysms [5,6]. 

Thiols are mercaptans consisting of sulfhydryl groups. They are mostly formed by albumin, proteins, and low-molecular weight molecules and switched to disulphide bonds under oxidative process through a reversible reaction. Abnormal thiol disulphide homeostasis is related to various cardiovascular diseases including coronary artery disease, primary hypertension, heart failure, and pulmonary hypertension and embolism [7-10]. When free oxygen radical-induced cell membrane damage occurs in cases of acidosis and hypoxia, serum albumin undergoes modification and its metal binding capacity decreases. This deformed albumin is called ischemia-modified albumin (IMA). It is an important biomarker for the identification of ischemic conditions such as coronary artery disease and oxidative stress-related diseases including diabetes mellitus (DM), hypercholesterolemia, and metabolic syndrome [11,12]. Ceruloplasmin, which is an extracellular major antioxidant molecule that prevents ferrous ion-induced lipid peroxidation through ferroxidase activity, was shown to be a marker of cardiovascular risk [13,14]. The role of oxidative stress parameters including thiol, disulphide, IMA, and ferroxidase activity in AAD development and their capability as a biomarker utilization is not well-known. In this study, we aimed to determine plasma thiol, disulphide, and serum IMA levels and ferroxidase activity in patients with AAD in comparison to those without AAD and to evaluate the predictive value of these oxidative stress markers for AAD.

## 2. Materials and methods

### 2.1. Patient sample and definitions 

This study was designed as a cross-sectional study of 184 consecutive patients who applied to our cardiology clinic from February 2018 to July 2018. Our study population consisted of patients with AAD (n = 85) and control group patients without AAD (n = 99). The inclusion criteria were as follows: being older than 18 years of age, accepting to participate in the study, and patients being diagnosed with AAD. The exclusion criteria were defined as patients with moderate to severe valve disease, prosthetic heart valve, bicuspid aortic valve morphology, an infectious disease, malignancy, pulmonary embolism, accompanying acute aortic and coronary syndromes, decompensated heart failure, more than mild hepatic or renal disease, and patients taking drugs with antioxidant effects. Additionally, patients with myocarditis, pericarditis, cardiomyopathy, Marfan syndrome, hepatotoxic drug use, biliary tract diseases, and secondary hypertension were excluded from the study.

The age, sex, medical history, cardiovascular risk factors, medications, physical examination findings, and basic laboratory analyses including complete blood count and standard biochemical parameters of all the patients were recorded electronically. Hypertension was defined as systolic blood pressure ≥140 mmHg or diastolic blood pressure ≥90 mmHg in at least 3 office measurements or as using antihypertensive therapy. DM was defined as blood glucose ≥126 mg/dl or as using antidiabetic drugs. Regardless of the amount, active smokers were defined as smoker. Body mass index (BMI) and body surface area (BSA) were calculated with the Mosteller formula.

The recommendations of the Good Clinical Practices Guidelines and the Declaration of Helsinki were followed during the study. 

### 2.2. Blood samples and laboratory measurements

Blood samples for the measurement of biochemical parameters were obtained at least after 8 hours of overnight fasting. Blood samples were taken from a cubital vein and biochemical parameter analyses were performed with Hitachi 747 autoanalyzer. The plasma samples were taken into EDTA/citrated tubes. On the other hand, serum samples were taken into tubes without EDTA/citrate. Plasma concentrations of total cholesterol, triglyceride, low-density lipoprotein (LDL) cholesterol, and high-density lipoprotein (HDL) cholesterol were calculated by enzymatic chemical cleaning method using Cobas 6000 (Roche Diagnostics GmbH, Mannheim, Germany).

A spectrophotometric method was used to determine the plasma thiol and disulphide levels as described previously by Erel et al. [15]. The serum IMA levels were measured automatically by a colorimetric method as described by Bar-Or et al. [16]. The serum ferroxidase activity was determined with a Hitachi 912 analyzer (Roche Diagnostics GmbH) according to the method described by Erel [17]. 

### 2.3. Echocardiographic measurements

All the participants underwent comprehensive two-dimen­sional, M-mode, and Doppler echocardiographic examinations performed by one experienced research echocardiographer, who was blinded to the study protocol. Echocardiographic examinations were performed by using a com­mercially available echocardiograph equipped with a 2.5- and 3.5-MHz transducer with Philips iE33 xMatrix (Philips Healthcare, Inc., Andover, MA). The left ventricular ejection fraction (LVEF) was calculated from the parasternal long axis images using the M-mode method. The left ventricular (LV) mass was measured using the Devereux formula [18]. The diameter of the ascending aorta (DAA) was measured by the leading-edge method from parasternal long-axis view in accor­dance with the European Society of Echocardiography guide­lines [19]. AAD was defined as DAA above 3.7 cm.

### 2.4. Statistical analysis 

The statistical analyses were carried out using IBM SPSS Statistics for Macintosh, Version 24.0 (IBM Corp., Armonk, NY, USA). The one-sample Kolmogorov–Smirnov test was used to evaluate the distribution of numerical variables. According to the results of this test, the Student’s t-test was applied to the numerical data which conforms to the normal distribution and the results were entered as mean and standard deviation. On the other hand, the Mann–Whitney U test, which is one of the nonparametric tests, was used for the skewed distributed variables. Considering the results of this test, the median and interquartile range values were used. The chi-square test was used for categorical variables. The Fisher’s exact test was applied in cases where the chi-square test could not be applied. For correlation analyses regarding AAD, the Pearson’s correlation analyses were preferred for data with normal distribution, otherwise Spearman’s correlation analyses were preferred for data with skewed distribution. Independent relationships of maximal aortic diameter were assessed by stepwise linear regression analyses. The variables which were significantly correlated with DAA and the variables known to be associated with AAD were included in the analyses. Afterwards, BMI, glomerular filtration rate (GFR), LVEF, and left atrium diameter (LAd) were applied into the first model. Hierarchically, the LV mass and the plasma total thiol levels were added into Model 2 and Model 3, respectively. The multicollinearity assessment was checked with correlation coefficient (r) and the variance inflation factor (VIF) value. Independent variables with a correlation coefficient (r) above 0.7 and a VIF value above 3 were considered to have multicollinearity and were not included in the same model. Receiver operating characteristic (ROC) curve analyses were used to determine the cut-off values for the sensitivity and specificity of the total thiol levels in predicting AAD. P values lower than 0.05 were considered to be statistically significant.

## 3. Results

Table 1 presents baseline characteristics and laboratory parameters including oxidative stress parameters of the study groups. Age, BMI, BSA, the presence of hypertension and DM, smoking status, glucose levels, LDL, triglyceride, neutrophil, lymphocyte, neutrophil to lymphocyte ratio (NLR), and thyroid stimulating hormone levels were similar between the groups. The total cholesterol and HDL levels and GFR were significantly higher in the control group (P = 0.04, P = 0.01, P < 0.001, respectively), whereas the creatinine levels were significantly lower in the control group compared to the AAD patient group (P = 0.001). The native thiol and the total thiol levels were significantly lower in the AAD group than the control group (372.1 ± 73.9, 439.2 ± 64.6, P < 0.001; 403.1 ± 64.1, 469.3 ± 64.1, P < 0.001; respectively). The other oxidative stress parameters such as disulphide, IMA levels, and ferroxidase activity were similar among the groups. 

**Table 1:** Baseline characteristics and laboratory parameters of the patients.

Variables	Normal ascending aortic diameter (n = 99)	Dilated ascending aortic diameter (n = 85)	p value
Age, years	65.1 ± 9.6	65.9 ± 9.0	0.60
Sex (male), n (%)	67 (67)	54 (53)	0.55
BMI (kg/m2)	28.1 (5.1)	29.4 (6.1)	0.06
BSA (m2)	1.9 (0.2)	1.89 (0.2)	0.75
Hypertension, n (%)	61 (61)	62 (72)	0.10
Diabetes mellitus, n (%)	30 (30)	29 (34)	0.58
Smoking, n (%)	24 (24)	21 (24)	0.94
Glucose (mg/dL)	102 (28)	105 (59)	0.32
Total cholesterol (mg/dL)	188.8 ± 42.5	175.1 ± 48.1	0.04*
HDL (mg/dL)	43 (12.2)	40.9 (18.3)	0.01*
LDL (mg/dL)	112.5 ± 37.7	101.7 ± 37.6	0.05
Triglycerides (mg/dL)	138 (116)	126 (96)	0.49
Creatinine (mg/dL)	0.82 (0.28)	0.94 (0.41)	0.001*
GFR (mL/min/ m2)	88 (26)	77 (39)	<0.001*
Neutrophil (K/uL)	4370 (1800)	4450 (2620)	0.88
Lymphocyte (K/uL)	2260 ± 776	2094 ± 859	0.17
NLR	2.07 (1.35)	2.1 (2.25)	0.26
TSH (uIU/mL)	1.5 (1.5)	1.4 (1.3)	0.36
Native thiol (µmol/L)	439.2 ± 64.6	372.1 ± 73.9	<0.001*
Total thiol (µmol/L)	469.3 ± 64.1	403.1 ± 64.1	<0.001*
Disulphide (µmol/L)	16.2 (12.7)	15.3 (13.3)	0.23
Disulphide/Native thiol	0.04 (0.03)	0.04 (0.03)	0.79
Disulphide/Total thiol	0.03 (0.03)	0.04 (0.03)	0.80
Native thiol/Total thiol	0.93 (0.06)	0.92 (0.06)	0.86
Ferroxidase (U/L)	465 (186)	498(210)	0.12
IMA (ABSU)	75.7 ± 4.3	75.2 ± 5.9	0.53

BMI: Body mass index; BSA: Body surface area; GFR: Glomerular filtration rate; HDL: High-density lipoprotein; IMA: Ischemic modified albumin; IQR: Interquartile range; LDL: Llow-density lipoprotein; NLR: Neutrophil to lymphocyte ratio; SD: Standard deviation; TSH: Thyroid stimulating hormone. Parameters were expressed as mean ± SD and median [IQR]. *P < 0.05 was considered significant for statistical analyses.

Table 2 presents the echocardiographic measurements of the study population. The patients with AAD had significantly lower LVEF values compared to the control group patients, whereas the LV end-diastolic diameter, LAd, the diameters of end-diastolic interventricular septum and posterior wall, and the LV mass and LV mass index were significantly higher in the AAD patients. 

**Table 2:** Echocardiographic measurements of the study population.

Variables	Normal ascending aortic diameter (n = 99)	Dilated ascending aortic diameter (n = 85)	P value
LVEDD (mm)	47 (4)	49 (7.5)	0.01*
Lad (mm)	39 (6)	43 (8)	<0.001*
LVEF (%)	60 (11)	55 (13)	0.001*
IVSd (mm)	12 (2)	12 (3)	0.002*
PWd (mm)	11 (2)	12 (1)	0.004*
LV mass (g)	204 (54)	234 (72)	<0.001*
LV mass index (g/m2)	105 (22)	123 (31)	<0.001*
Ascending aorta (mm)	34 (4)	41 (3)	<0.001*

IQR: Interquartile range; IVSd: End-diastolic interventricular septum diameter; Lad: Left atrium diameter; LV mass: Left ventricle mass; LVEDD: Left ventricle end-diastolic diameter; LVEF: Left ventricle ejection fraction; PWd: End-diastolic posterior wall diameter; SD: Standard deviation. Parameters were expressed as mean ± SD and median [IQR]. *A P value less than 0.05 was considered significant for statistical analyses.

Table 3 shows the correlation analyses of DAA with various variables. The native thiol and the total thiol levels were significantly and inversely correlated with DAA (r = –0.38, P < 0.001; r = –0.39, P < 0.001; respectively) (Figure). In a similar manner, GFR and LVEF were inversely correlated with DAA with statistical significance. On the other hand, BMI, the LV mass, and LAd were positively and significantly correlated with DAA.

**Table 3:** Ascending aortic diameter and their correlation with variables.

Variables	Ascending aortic diameter	
	r coefficient	P value
Age	0.09	0.21
GFR	–0.24	0.001*
BMI	0.15	0.03*
Glucose	0.07	0.32
Total cholesterol	–0.11	0.11
HDL	–0.14	0.05
LDL	–0.11	0.13
Triglycerides	–0.01	0.85
NLR	0.12	0.08
LVEF	–0.19	0.008*
LV mass	0.37	<0.001*
LAd	0.35	<0.001*
Native thiol	–0.38	<0.001*
Total thiol	–0.39	<0.001*
Disulphide	–0.09	0.18
Ferroxidase	0.13	0.06
IMA	–0.008	0.91

BMI: Body mass index; GFR: Glomerular filtration rate; HDL: High-density lipoprotein; IMA: Ischemic modified albumin; Lad: Left atrium diameter; LV mass: Left ventricle mass; LVEDD: Left ventricle end diastolic diameter; LVEF: Left ventricle ejection fraction; LDL: Low-density lipoprotein; NLR: Neutrophil to lymphocyte ratio. *A P value less than 0.05 was considered significant for statistical analyses.

**Figure:**
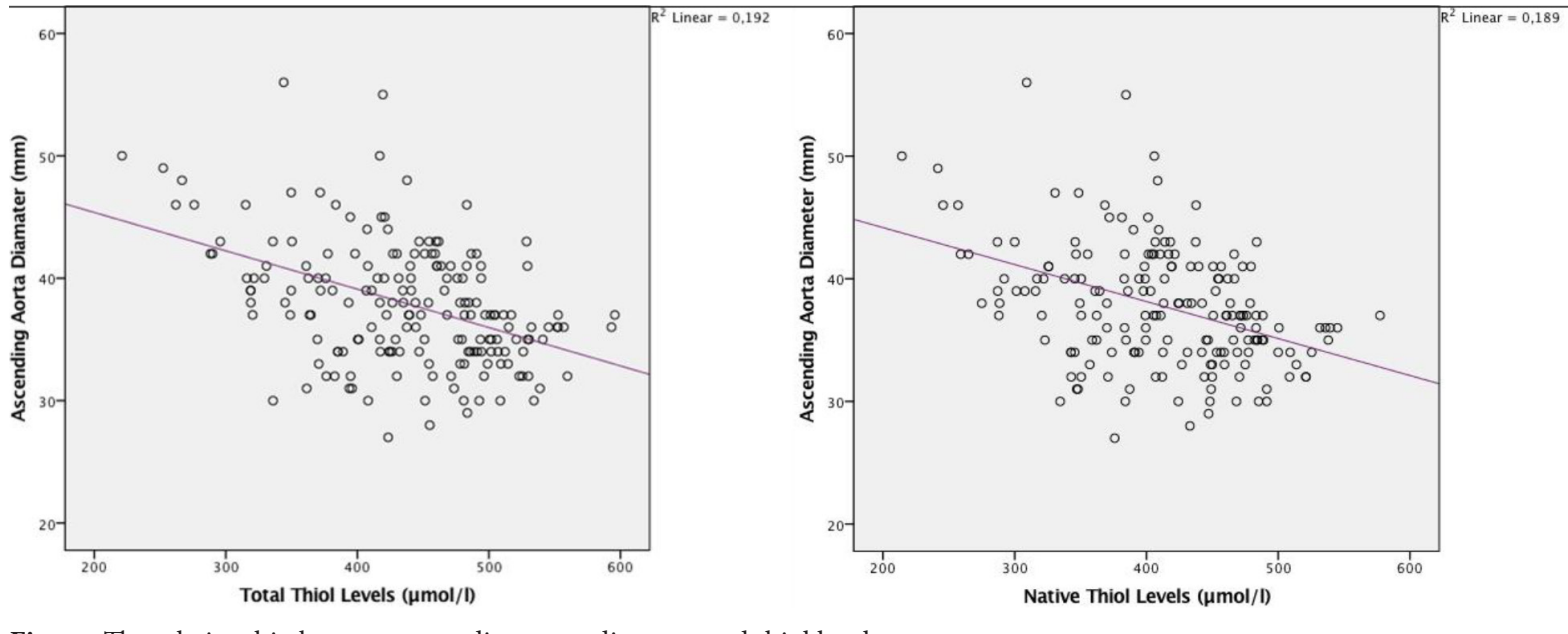
The relationship between ascending aorta diameter and thiol levels.

Stepwise linear regression analysis, which is also given in Table 4, showed that the LV mass and the plasma total thiol levels are independent predictors of DAA (β = 0.223, P = 0.02; β = –0.340, P < 0.001, respectively). The area under the ROC curve for the total thiol level predicting AAD was 0.755 (95% CI: 0.687–0.823, P < 0.001). The cut-off value of the total thiol level (436.3) was associated with 69% sensitivity and 62% specificity for AAD.

**Table 4:** Stepwise linear regression analyses for ascending aortic diameter.

Variables	Model I Adjusted R^2^ = 0.115			Model II Adjusted R^2^ = 0.160 R square change: 0.05			Model III Adjusted R^2^ = 0.243 R square change: 0.09		
	Standardized β	P value	Collinearity statistics (VIF)	Standardized β	P value	Collinearity statistics (VIF)	Standardized β	P value	Collinearity statistics (VIF)
LVEF	–0.097	0.20	1.22	-0.052	0.49	1.26	–0.002	0.98	1.29
GFR	–0.205	0.01*	1.20	–0.174	0.02*	1.11	–0.041	0.34	1.32
LAd	0.166	0.03*	1.09	0.092	0.23	1.31	0.071	0.58	1.32
BMI	0.136	0.06	1.04	0.122	0.08	1.04	0.085	0.20	1.06
LV mass				0.249	0.001*	1.25	0.223	0.002*	1.26
Total Thiol							–0.340	<0.001*	1.36

Stepwise linear regression analysis. The regression model included body mass index (BMI), glomerular filtration rate (GFR), left atrium diameter (LAd), left ventricular ejection fraction (LVEF, %), left ventricle mass (LV-mass) and total thiol as possible independent variables. Standardized β: Beta c oefficient; VIF: Variance inflation factor.

## 4. Discussion

In this study, the plasma total thiol and the native thiol levels, which are the indicators of oxidative stress, were lower in the AAD patients compared to the control group patients with normal DAA. In addition, the plasma total thiol level and the LV mass were demonstrated to be distinctive factors of DAA. However, there was no difference in disulphide and IMA levels or ferroxidase activity between the groups. 

Oxidative stress is defined as tissue damage occurring secondary to increased production and/or decreased destruction of reactive oxygen species (ROS). The nicotinamide adenine dinucleotide phosphate oxidase and xanthine oxidase pathways are responsible for vascular oxidative stress [3]. Beyond traditional risk factors for aortic aneurysms such as advanced age, hypertension, and smoking, MMPs and ROS are also considered to be important risk factors in the pathophysiology of AAD [20,21]. In a recent study published by Akkuş et al., the native thiol and the total thiol levels were demonstrated to correlate moderately with maximal aortic diameter. The total thiol level was also found to be an independent predictor of maximal aortic diameter in this study, and it was observed that the thiol levels increased after surgical repair of the aorta [1]. Likewise, the total and native thiol levels were lower in the patients with AAD in our study. Our study group consisted of a relatively high number of patients when compared to the study of Akkuş et al., and the patients in our study tended to be older, which is a significant contributor to oxidative stress. We also investigated the role of disulphide, IMA levels, and ferroxidase activity in our population, although there was no difference between the groups. We used transthoracic echocardiography to evaluate the ascending aorta, and AAD was defined as a DAA above 3.7 cm. However, Akkuş et al. applied computed tomography in their study, and they included patients with thoracic aorta enlargement above 4.0 cm. Due to our study design, we did not investigate whether the treatment improved thiol levels, and we also excluded patients with acute aortic syndrome.

Unlike previously published studies, we investigated the role of other oxidative stress parameters, including disulphide, IMA levels, and ferroxidase activity. However, these parameters were comparable among the groups. There are no studies in the literature investigating the relationship between aortic pathologies and ferroxidase activity, and data evaluating the IMA levels in this patient group are limited and have contradictory results. A previous study reported that there was no difference between the IMA levels in the aortic dissection and aneurysm groups compared to the healthy control group [22]. Conversely, a study by Eroğlu et al. demonstrated that the IMA levels were higher in the aortic aneurysm group than the control group [23]. These contradictory results may be due to the differences in number, age, demographic characteristics, sex, and race of the patients included in the studies. In our study, we did not observe any significant difference regarding the serum IMA levels between the AAD group and the control group. There may be several reasons for comparable IMA levels while thiol levels are altered in AAD patients. The first may be related to the different rates of biochemical kinetics of these oxidative stress parameters. There are no data on the time periods required for changes in thiol-disulphide homeostasis and ferroxidase activity. In studies of coronary artery disease, the IMA levels increased immediately after exposure to ischemia and decreased to normal levels within 12 hours [22]. Another reason that may explain this difference may be the rate of exposure to physiological variables, which significantly affects the IMA levels [23]. Finally, it may be necessary to have more severe oxidative damage, such as ischemia, acidosis, or tissue hypoxia, in order to change the IMA and ferroxidase activity. In the case of AAD, the oxidative process is a progressive condition that spreads over a longer period of time, but to a lesser degree compared to acute conditions. 

There are various limitations of our study. Our study includes a relatively small population recruited from a single center. Large-scale epidemiological studies conducted over several years are required to correlate the cross-sectional findings from this study with clinical outcomes. The association between AAD and various oxidative stress markers, such as uric acid, gamma-glutamyl transferase, asymmetric dimethylarginine, alpha-1 antitrypsin, C-reactive protein, interleukin-6, and NLR, has previously been shown by different methods in the literature [2, 24–29]. Although there was no difference between the groups in terms of NLR in our study population, we did not evaluate all of the oxidative stress parameters. In addition, only the transthoracic echocardiography measurements were used as diagnostic imaging methods. Computed tomography, magnetic resonance imaging, and transesophageal echocardiography measurements can detect aortic aneurysms in other anatomic locations and improve the accuracy of ascending aortic measurements. Lastly, low thiol levels may not only be a suspicious precipitating factor of aortic enlargement, but also a by-product of dilatation.

In conclusion, among oxidative stress parameters including thiols, disulphide, IMA, and ferroxidase activity, only lower total thiol levels appear to confer a high risk for AAD development. Along with the proven diagnostic imaging methods, thiol levels may be helpful to diagnose and stratify patients with AAD. Pathophysiological and biochemical mechanisms of oxidative stress during AAD development may be identified with the help of future studies.

## Acknowledgement/Disclaimers/Conflict of interest

This research received no grant from any funding agency in the public, commercial or not-for-profit sectors.

## Informed consent

Approval of the Ethics Committee of Yıldırım Beyazıt University Faculty of Medicine has been obtained for this study (26379996/52). Particular data regarding medical record, age, and sex have been obtained from the participants as per their written informed consent.
